# Chikungunya Virus 3′ Untranslated Region: Adaptation to Mosquitoes and a Population Bottleneck as Major Evolutionary Forces

**DOI:** 10.1371/journal.ppat.1003591

**Published:** 2013-08-29

**Authors:** Rubing Chen, Eryu Wang, Konstantin A. Tsetsarkin, Scott C. Weaver

**Affiliations:** 1 Center for Tropical Diseases and Department of Pathology, University of Texas Medical Branch, Galveston, Texas, United States of America; 2 Department of Microbiology and Immunology, University of Texas Medical Branch, Galveston, Texas, United States of America,; Institut Pasteur, France

## Abstract

The 3′ untranslated genome region (UTR) of arthropod-borne viruses is characterized by enriched direct repeats (DRs) and stem-loop structures. Despite many years of theoretical and experimental study, on-going positive selection on the 3′UTR had never been observed in ‘real-time,’ and the role of the arbovirus 3′UTR remains poorly understood. We observed a lineage-specific 3′UTR sequence pattern in all available Asian lineage of the mosquito-borne alphavirus, chikungunya virus (CHIKV) (1958–2009), including complicated mutation and duplication patterns of the long DRs. Given that a longer genome is usually associated with less efficient replication, we hypothesized that the fixation of these genetic changes in the Asian lineage 3′UTR was due to their beneficial effects on adaptation to vectors or hosts. Using reverse genetic methods, we examined the functional importance of each direct repeat. Our results suggest that adaptation to mosquitoes, rather than to mammalian hosts, is a major evolutionary force on the CHIKV 3′UTR. Surprisingly, the Asian 3′UTR appeared to be inferior to its predicted ancestral sequence for replication in both mammals and mosquitoes, suggesting that its fixation in Asia was not a result of directional selection. Rather, it may have resulted from a population bottleneck during its introduction from Africa to Asia. We propose that this introduction of a 3′UTR with deletions led to genetic drift and compensatory mutations associated with the loss of structural/functional constraints, followed by two independent beneficial duplications and fixation due to positive selection. Our results provide further evidence that the limited epidemic potential of the Asian CHIKV strains resulted from founder effects that reduced its fitness for efficient transmission by mosquitoes there.

## Author Summary

The 3′ untranslated genome region (UTR) of arbovirus (arthropod-borne virus) RNA genomes is characterized by enriched direct repeats (DRs) and stem-loop structures, which are thought to play a specific role in maintaining efficient transmission in multiple hosts. However, this hypothesis is vague and has little experimental support. Based on our observation of a distinct, lineage-specific sequence structure in the chikungunya virus (CHIKV) Asian lineage 3′UTR, we tried to understand the underlying driving forces on the arboviral 3′UTR evolution. Specifically, we sought to determine whether the dramatic genetic changes in the Asian lineage 3′UTR were the result of adaptation into the new host or vector populations. Using reverse genetic methods, we obtained results that suggested that the DRs have a significant effect on the viral fitness level in mosquitoes but not in mammals. Interestingly, instead of a directional selection from its ancestral state, our results suggest that the evolution of the CHIKV Asian lineage 3′UTR involved a population bottleneck with a deleterious deletion of DRs, followed by accumulation of mutations due to the loss of structural/functional constraints. Later, the duplication of two regions that are beneficial in mosquitoes led to the fixation of this 3′UTR sequence in Asian lineage, possibly facilitating the 1958 Thailand outbreak.

## Introduction

Genetic change, which can lead to adaptation to new hosts or vectors, is a major cause of the emergence or re-emergence of arthropod-borne viral (arboviral) and other RNA viral diseases [1], [2]. However, compared to the numerous investigations of point mutations within viral genomic open reading frames, the evolution and determinants of fitness of untranslated genome regions (UTRs) have received far less attention. The 3′ UTRs of arboviral genomes exhibit large size variations, ranging from ∼100 nt to more than 700 nt, and involving extensive substitutions, insertions and deletions even within viral species. This length variation suggests that the heterogeneous regions may not be essential for replication, a view supported by experimental studies with genetically engineered viruses lacking a large part of the 3′UTR that remain viable, albeit with different levels of attenuation [3]–[8]. However, these seemingly redundant sequences must play some role favored by natural selection, because otherwise longer genomes should theoretically be less efficiently replicated. Improved understanding of the forces driving the evolution of the arboviral 3′UTR is needed to provide important insights on its role on pathogenesis and host/vector adaptation.

An interesting observation is that the variable region in the 3′UTR often contains direct repeats (DRs) in the arboviral genera *Alphavirus* [9], [10] and *Flavivirus* [11]. These DRs can be relatively conserved in closely related viruses, indicating that repeat duplication may serve as a major evolutionary mechanism for the 3′UTR, and that these DRs may have functional significance. Indeed, sequence comparisons of flaviviruses suggest that duplication of long repeat elements (LREs) and extensive deletions are the main evolutionary mechanisms of the 3′UTR [12], [13]. Despite their high level of sequence diversity, secondary structure predictions suggest that the flavivirus 3′UTR comprises enriched stem-loop structures, with some conserved structural motifs observed in all species, suggesting functional selection [14]. Furthermore, sequence comparisons of different eco-groups suggest that mosquito-borne flaviviruses, which usually use multiple invertebrate and/or vertebrate host species, have a more diverse 3′UTR than tick-borne or non-vector-borne flaviviruses, which have more limited host ranges and/or transmission dynamics [11]. This raises the hypothesis that the DRs may interact with host/vector factors to maintain efficient replication in multiple hosts, and to facilitate the adaptation to new hosts.

An interaction between viral 3′UTR and host proteins has been indicated by several kinds of data. First, the 3′UTRs in both alphaviruses [15]–[18] and flaviviruses [19] interact with cellular proteins (in both mosquito and mammalian cells) to directly or indirectly facilitate genome replication. It has been observed that several alphaviruses usurp the cellular HuR protein, which enhances mRNA stability and therefore inhibits viral RNA decay [16]–[18]. Additionally, arboviral 3′UTRs can encode microRNAs (miRNAs, such as observed in West Nile virus) which regulate cellular gene expression to enhance viral replication [20]. Finally, flaviviruses generate one or more small subgenomic flavivirus RNAs (sfRNAs), which are essential for pathogenicity in vertebrate cells and in mice [21]. These sfRNAs are collinear with the 3′UTR and produced by incomplete viral RNA degradation by the host 5′-3′ exonuclease XRN1, mediated by pseudoknot (PK) structures upstream of the 3′UTR [22], [23]. Their observed functions include: 1) regulation of antigenome synthesis [24], 2) inhibition of the cellular exoribonuclease XRN1 and alteration of host mRNA stability [25], 3) evasion of the Type I Interferon response [26], and 4) RNA inference suppression in both mammalian and insect cells by inhibiting Dicer-mediated in vitro cleavage of double-stranded RNA [27].

**Figure 1:**
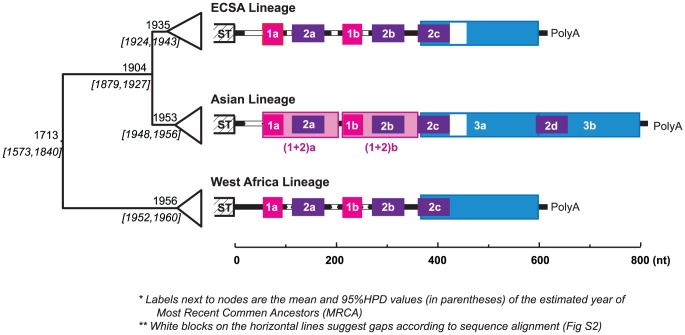
Evolution history and lineage-specific structures of the CHIKV 3′UTR. On the left is the MCC (Maximum Clade Credibility) tree based on the complete ORF sequences, with the branches in each lineage collapsed. The estimated year of the most recent common ancestor (MRCA: mean and the 95% HPD values) of each clade is labeled left to the node. The 3′UTR structures, based on sequence alignment, are shown next to each lineage. Direct repeats are illustrated by different colored blocks, each of the four colors represents a different homologous sequence region. Sequence gaps in the alignment are indicated by white blocks. In the Asian lineage, two distinct derived differences are observed: 1) duplication of DR3, and duplication the of DR(1+2) region. The detailed alignment can be found in Fig. S2.

Despite these advances in understanding the functional roles of arboviral 3′UTRs, there is no solid evidence to relate the occurrence of indels, which appear to occur frequently during their evolution, with any particular adaptation to a given host or vector. The extensive within-species diversity in the 3′UTR of the alphavirus chikungunya virus (CHIKV), especially lineage-specific DR patterns (revealed in this study; Fig. 1), is unique within this genus of mainly mosquito-borne viruses. Together with prior reconstructions of CHIKV evolutionary history [28], as well as the relative sequence conservation and comparability among lineages, this diversity in 3′UTR sequences provides a unique opportunity for understanding their evolution and functional importance.

Chikungunya virus (*Togaviridae*: *Alphavirus*) is transmitted among nonhuman primates and humans via *Aedes* spp. mosquitoes. It causes chikungunya fever, a febrile illness associated with debilitating arthralgia and rash [29]. Chikungunya virus has a single-stranded, positive sense RNA genome of ∼12 kb, including a notably long 3′UTR ranging from ∼500 to 700 nt. Enzootic in tropical and subtropical regions of Africa, CHIKV has emerged several times into a human-mosquito urban cycle to cause major epidemics both within and outside of Africa. Phylogenetic analyses suggest that the currently circulating CHIKV strains form three major geographic lineages, namely the entirely enzootic West African lineage, the East, Central and South African (ECSA) enzootic lineage, which includes the recently emerged epidemic strains responsible for Indian Ocean basin and Asian outbreaks, and the Asian lineage [28], which has been circulating in an *Aedes aegypti*-human cycle for over 50 years. Interestingly, CHIKV genome comparisons suggest lineage-specific 3′UTR structures, with the Asian lineage exhibiting a unique pattern of mutation, duplication, and insertion (Fig. 1). Although the most recent common ancestor of the Asian lineage is estimated to have occurred in the early 1950s, just before the 1956 Thailand outbreak, it is not clear when this lineage was introduced from eastern Africa into Asia or whether the distinct mutations and structural rearrangement in the Asian 3′UTR occurred before or after this introduction and establishment of the urban cycle. It is also unknown whether this novel Asian 3′UTR structure is the result of adaptation to the urban transmission cycle there [Although CHIKV antibodies have been detected in nonhuman primates in Asia [30], spillback from human transmission cycles is difficult to rule out] such as to the urban vector *A. aegypti* implicated in all Asian outbreaks prior to 2007.

To address these questions related to evolution of the 3′UTR and its potential influence on the epidemic potential of the Asian CHIKV strains, we dissected the inferred structural changes in the 3′UTR of the Asian CHIKV lineage and explored their effects on the replication in vertebrate hosts and vectors. Our findings provide important insights into the functional role of the mosquito-borne arbovirus 3′UTR.

## Results

### Lineage-specific Direct Repeat structure in the CHIKV 3′UTR

To explore the repeat structure in the CHIKV genome, DNA matrix comparisons were conducted based on representative strains from each of the three major lineages (West Africa, ECSA and Asian). The results suggested that the 3′UTR, but not other genome regions, contains multiple DRs, with the Asian lineage having a distinct pattern (Fig. S1). Imposing these repeat patterns onto the rough sequence alignment generated from the guide tree based on the complete open reading frame sequences led to a refined and reliable alignment with striking lineage-specific structures and minor indels within each lineage. The complete sequence alignment is available upon request, with a simplified version shown in Figure S2. As illustrated in Fig. 1, the CHIKV 3′UTR contains two DR elements consistent in the West African and ECSA lineages, namely DR1 (39 nt, two copies) and DR2 (62 nt, 3 copies). However, the Asian 3′UTR is distinct, including 1) a long insertion (193 bp) near the 3′ end, which is the result of the direct duplication of its 5′-adjacent region (Fig. 1, shaded blue), and 2) accumulated mutations (point mutations and insertions) around DR2a, including the DR1a region, and the duplication of this entire region [hereafter designated as DR (1+2)] to replace the DR1b/DR2b region, or vice versa. Previous studies annotated the alphavirus 3′UTR into three repeat sequence elements (RSEs) and a 19 nt conserved sequence element (CSE) at the 3′ end [9]. The DR2 found in our study corresponds to RSEs defined previously [18]. However, DR1 and DR3 have not been described previously, and we found the 19 nt CSE is not strictly conserved, with occasional mutations that change its length observed among sequences. Interestingly, DR3 is immediately adjacent to the 19 nt CSE which is believed essential in viral replication [31].

To determine if these DRs form any structural/functional units, as suggested in flaviviruses [11], [14], RNA secondary structures were predicted via Mfold [32], STAR [33], and Vienna [34], [35], at both 37°C (typical primate host temperature) and 28°C (typical mosquito vector temperature in the tropics). These programs generated slightly different secondary structures (data not shown). In most predictions the folds of different copies of DR1 and DR 2 differed, and the overall structures generally differed at 28° and 37°C (except those estimated by Vienna). Figure S3 shows a sample of top ranked structures produced by Mfold. In summary, there are many short stem-loop structures distributed throughout 3′UTR, and they form the basis for a higher-level secondary structure. Despite the many stem-loop structures in DR1 and DR2, the repeating elements themselves may not necessarily correspond to a specific uniform structure. This interpretation is consistent with previous analyses of flaviviruses [11]. According to Mfold, this region may also fold into different structures at 37°C and 28°C, with the former more compact and the latter looser. In contrast, the DR3 region is relatively conserved and contains a distinct Y-shaped structure of 80 nt (Fig. S3, dark blue) conserved in all three CHIKV lineages at both 37°C and 28°C, suggesting its functional importance. Notably, duplication of DR3 in the Asian lineage added another copy of this Y-shaped structure.

### 3′UTR differentially affects replication kinetics in C6/36 versus Vero cells

**Figure 2:**
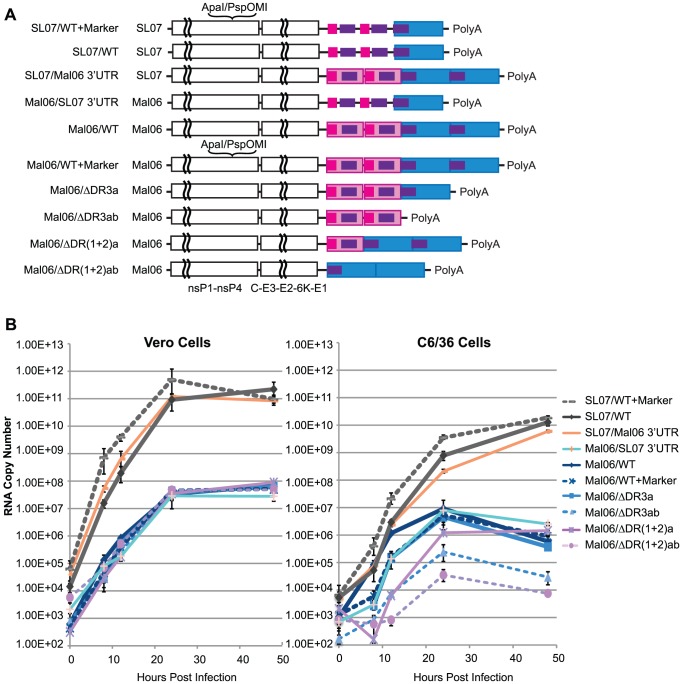
Replication kinetics of CHIKV variants in Vero and C6/36 cells. A. Genome structures of wt and genetic engineered CHIK viruses based on Mal06 (Asian lineage) and SL07 (ECSA lineage) used in this study. B. Replication kinetics of these CHIKV variants in Vero and C6/36 cells. Cells were infected in triplicate by different CHIKV variants in MOI = 0.1. RNA copy number at selected time points post infection was measured by Real-Time RT-PCR. Error bars show the maximum and minimum value in the triplicates.

To evaluate the effect of the 3′UTR on CHIKV replication, we engineered a series of mutant viruses based on two wild-type (wt) CHIKV strains, the Mal06 strain (MY002IMR/06/BP; GenBank Acc. No. EU703759.1), a representative of the Asian lineage, and the SL07 strain (SL-CK1; GenBank Acc. No. HM045801.1), representing the ECSA lineage. Fig. 2A illustrates the genetic organization of the engineered viruses, including 1) the wt Mal06 and SL07, 2) modified version of each including synonymous mutations as genetic markers, 3) chimeras of each strain with swapped 3′UTRs, and 4) Mal06 variants with either 1 or 2 copies of DR(1+2) or DR3 deleted. Their fitness levels were first compared through replication kinetics in both Vero (African green monkey) and C6/36 (*A. albopictus*) cells, and then further evaluated through competition tests *in vivo*.

Comparing the replication kinetics of these CHIKV strains provided interesting insights into the role of the 3′UTR in mammalian and insect cells (Fig. 2B). First, viruses from both the ECSA and Asian lineages replicated rapidly, reaching peak titers 24–48 h post-infection. Variants derived from the ECSA SL07 strain showed significantly (∼3–4 log difference) higher replication in both C6/36 and Vero cells. These results were consistent with the observation that the IOL lineage strains have been rapidly replacing the local Asian lineage strains in Southeast Asia since 2006 [36]. Our finding that chimeric viruses with 3′UTRs derived from a different lineage exhibit no significant fitness change in Vero cells and only a slight fitness change in C6/36 also suggested that the ORF, rather than the 3′UTR, is the main determinant of CHIKV replication efficiency. Strikingly, the Asian Mal06 strain variants with swapped 3′UTRs exhibited significantly different replication kinetics in C6/36 cells, with RNA copies per ml ranging from 8.2×10^2^ [Mal06 with deletion of 2 copies of DR(1+2)] to 1.2×10^6^ (wt) at 12 h post-infection, and 3.5×10^4^ [Del-DR(1+2)ab] to 9.2×10^6^ (wt) at 24 h post-infection, respectively. In contrast, no significant difference was observed for any ML06 variants at either 24 (2.4–4.7×10^7^ copies/mL) or 48 h post-infection (4.8–8.9×10^7^ copies/mL) in Vero cells, indicating that the CHIKV 3′UTR may play a more important role in interacting with cell factors in mosquito than mammalian hosts. These results also suggest that genetic variation in the 3′UTR does not have a major effect on its interaction with viral proteins and/or RNA.

Consistent with reports from other arboviruses [3]–[8], CHIKV with only a partial 3′UTR was still viable in cell cultures, but with altered replication kinetics, especially in mosquito cells. In C6/36 cells, deleting either one or both copies of DR(1+2) led to a significant reduction in the replication rate, with the deletion of both copies having the most severe effect. These data indicate that the role of DR(1+2) does not rely on the presence of two copies, such as in the formation of a dimer proposed previously [12].

Similarly, deleting both copies of DR3 in the Asian Mal06 strain also led to a severe reduction in its replication rate in mosquito cells (Fig. 2). The fitness loss from deletion of both DR3 copies was intermediate between that resulting from deleting one copy of DR(1+2) and deleting both DR(1+2) copies. Interestingly, deleting only one copy of DR3 from the Mal06 strain did not significantly change its replication kinetics in C6/36 cells.

### Competition tests to assess fitness effect of DRs and to compare the Asian and ECSA 3′UTRs

**Figure 3:**
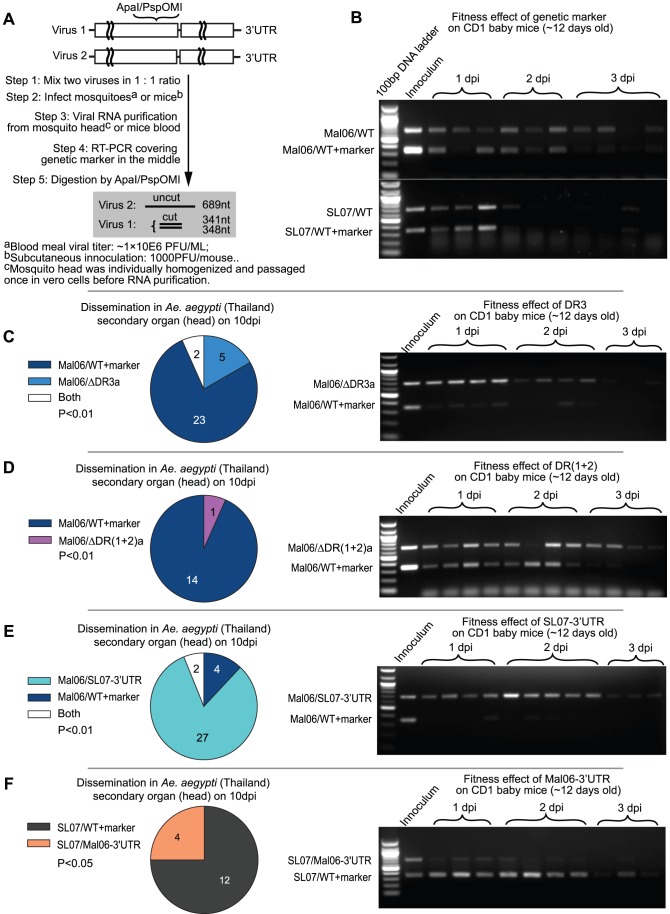
Competition tests on mosquitoes and mice. A. Experimental design. Competing viruses (one of them contains a synonymous genetic marker) were mixed in a 1∶1 initial ratio based on genome copies, and used for mosquito and mice infection. The viral RNA ratio was reflected by RT-PCR amplification of a region containing the marker in the middle, followed by thorough digestion on the digestion sites created by the genetic marker. In agarose gel analyses, the lower band reflects the level of virus with the genetic marker, whereas the upper band reflects the RNA level of virus without the genetic marker. B. Competition results between the two wt viruses (Mal06 and SL07) and their correspondent mutants in CD1 baby mice. C–F. Competition between 4 pairs of viruses (C: Mal06/ΔDR3a vs. Mal06/WT+marker; D: Mal06/ΔDR(1+2)a vs. Mal06/WT+marker; E: Mal06/SL07-3′UTR vs. Mal06/WT+marker; F: SL07/Mal06-3′UTR vs. SL07/WT+marker) on the dissemination rate in *A. aegypti* (Thailand) and viral RNA level in CD1 baby mice. The mosquitoes were infected through blood meal with viral titer in ∼1×10^6^ pfu/ml. On day 10 post infection, the heads of mosquitoes were dissected to study the viral dissemination. The numbers of samples infected by each virus are shown by pie graph, with statistical significance assessed using a Chi-square test. Viruses are labeled in the same colors as in Fig. 2B. CD1 baby mice were infected with initial dose of 1×10^4^ pfu, 3 or 4 of them were sacrificed each day and blood viral ratio was used to measure the fitness level of competing viruses (shown in the gel).

Due to the intrinsic experimental error caused by 1) small variations in the viral titer of initial inocula, 2) variation in cell density among triplicate test samples, and 3) RNA quantification, competition tests were conducted to more sensitively compare fitness levels between virus pairs. To determine the underlying reason for the fixation of Asian lineage CHIKV 3′UTR, we evaluated its fitness for infection and dissemination in the main Asian mosquito vector (1950s–2007 during evolution of the Asian lineage), *A. aegypti*, and viremia in the surrogate vertebrate host, CD1 mice infected at 11–12 days of age [37] using competition experiments. The Mal06 variants containing a deletion of one copy of either DR(1+2) or DR3 were competed against the genetically marked wt strain, and the results are shown in Fig. 3. Control competitions indicated that the synonymous genetic marker did not significantly influence viral fitness in mosquitoes [1] or CD1 mice (Fig. 3B).

Strikingly, deleting DR3a in the Asian Mal06 strain produced contrasting effects in mosquito versus mammalian hosts. In mosquitoes, only wt virus was detected from the majority (23/30) of the mosquito heads 10 dpi after a mixed bloodmeal, whereas virus with only one copy of DR3 was found only in 5/30 samples, with two of them showing a mixture of both competitors (Fig. 3C). In contrast, Mal06 with a deletion of DR3b outcompeted the wt virus in CD1 mice, as indicated by virus ratios in all the blood samples taken 1–2 days post-infection (dpi; Fig. 3C). This result was consistent with our hypothesis that the direct effects of the 3′UTR can outweigh the potential detrimental effects of genome length to determine CHIKV fitness.

Similarly, deleting one copy of the DR(1+2) led to a significant CHIKV fitness loss in mosquitoes, and a slight advantage in mice. Specifically, following mosquito infection and dissemination, and assay at 10 dpi, 14 of 15 infected mosquito heads contained only the wt Mal06 virus, whereas only one was infected by the mutant [Mal06/ΔDR(1+2)a] (Fig. 3D). Despite the nearly equal RNA ratio between the two viruses during the first two days after infection of mice, the mutant virus with only one copy of DR(1+2), and thus shorter genome, showed a significantly higher prevalence at 3 dpi, indicating its selective advantage at later stages of infection (Fig. 3D). The less dramatic effect of deleting DR(1+2)a compared to DR3a may be due to its shorter length (155 vs. 193 nt). In addition, the initial inoculum ratio of Mal06/ΔDR(1+2)a was slightly lower in the competition test in CD1 mice, which may also have influenced the outcome. In conclusion, retaining two copies of DR(1+2) provided a selective advantage to CHIKV in mosquitoes but not in vertebrates, compared to only one copy.

Despite the selective advantage for the infection and dissemination in mosquitoes of having two DR3 copies, it is not clear whether the fixation of the current CHIKV Asian lineage 3′UTR was due to an improved fitness level compared to its ECSA ancestor, which parsimony analysis predicted shared the 3′UTR seen in extant ECSA strains. To address this question, chimeric viruses were generated with backbones from the Mal06 strain (Asian lineage) and SL07 strain (ECSA lineage) and swapped 3′UTRs, and their relative fitness levels were compared using competition tests. Surprisingly, the chimeric virus Mal06/SL07 3′UTR outcompeted the Mal06 wt strain in both mosquitoes and mice (Fig. 3E). In contrast, the chimeric virus with the SL07 backbone and the Mal06 3′UTR exhibited lower fitness than its wt ECSA counterpart (Fig. 3F).

## Discussion

### Evolution path of the CHIKV Asian lineage 3′UTR

Investigations of the function and evolution of 3′UTRs in arboviruses, including repeat identifications [9], [10], [13] and secondary structure predictions [12], [14], [38], as well as experimental studies [3]–[8], [39], [40], have taken place for many years. However, ongoing positive selection on the 3′UTR has never been observed in ‘real-time,’ and the role of the 3′UTR remains poorly understood. A distinct 3′UTR sequence pattern was observed in all Asian lineage CHIKVs sampled from 1958 to 2009. As shown in Fig. 1, this Asian lineage 3′UTR contains an insertion of 193 nt at the 3′ end, the result of a direct duplication of the 5′end of the adjacent UTR region. In addition, both copies of direct repeats of DR(1+2) contain the same accumulated mutations found in the corresponding ECSA and West African UTRs. No intermediate 3′UTR form has been observed in CHIKV sequences from other lineages, raising the interesting questions: When and what caused the unique pattern of the Asian 3′UTR, and why did it become fixed in Asia? Was the current Asian lineage 3′UTR formed before or after its introduction into Asia? If it was formed before the introduction, why has it apparently disappeared from Africa? Or if it was formed after the introduction into Asia, what fitness advantage did it provide over the ancestral UTR?

Our results offer some insights into these questions. First, the 3′UTR from the ECSA lineage has significantly higher fitness than that of the Asian lineage in both *A. aegypti* and mice when placed into either the ECSA or Asian genetic backbone. This suggests that the fixation of the Asian 3′UTR was not due to an increased fitness level compared to its ancestor, and was not likely a result of directional (positive) selection. Next, the duplication of the Asian DR3 imparts increased fitness in mosquitoes, indicating its selective advantage in transmission. Similarly, the deletion of one copy of the DR(1+2) region leads to reduced fitness in mosquitoes but slightly higher fitness in mice. Finally, in contrast to the compact stem-loop structures observed in West African and ECSA 3′UTRs, part of the Asian DR(1+2) region contains a fragment of linear sequence that is not predicted to form a stable stem-loop structure, indicating that the mutations in the DR(1+2) region may have been tolerated due to its lack of or loss of a structural/functional constraint.

**Figure 4:**
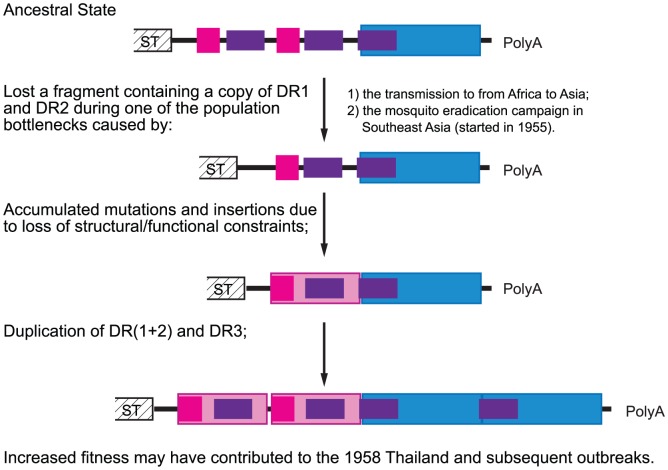
Hypothetical evolutionary pathway of CHIKV Asian lineage 3′UTR. Color blocks in this figure correspond to those in Fig. 1.

Based on this, we propose an evolutionary path of the Asian CHIKV 3′UTR illustrated in Fig. 4. First, a deletion occurred that resulted in the loss of one copy each of DR1 and DR2. Compared to its inferred ECSA ancestor, this mutant strain was presumably debilitated in its fitness for infection and dissemination in its principal vector, *A. aegypti* although it may have had a slight fitness increase for replication in humans based on the murine model. In the large enzootic CHIKV populations that exist in Africa, this mutant could disappear quickly due to its low frequency. The rapid fixation of such a mutant in Asia can only be explained by a population bottleneck where stochastic events can facilitate the fixation of a beneficial allele, and even allow a mutant with reduced fitness to circumvent selection. It is possible that this mutation accompanied the intercontinental transmission from Africa to Asia, which probably involved one or a few infected persons; it is also possible that a CHIKV population bottleneck was influenced by a mosquito eradication campaign in Southeast Asia (1955–1969). Although this effort was designed to eliminate malaria [41], [42], it included the use of DDT inside homes, which also reduced populations of *A. aegypti* responsible for urban CHIKV transmission. The use of DDT was also instrumental in the eradication of *A. aegypti* in many parts of the Americas during the 1950s to1960s [43], [44]. The coincidence of our estimated year of the most recent common ancestor of currently circulating Asian CHIKV lineage (1948–1956) with this malaria eradication campaign suggests a possible link.

Second, due to the breakdown of previous structural/functional constraints on the now deleted 3′UTR region, many neutral mutations accumulated in the DR (1+2) region of the Asian lineage. Because the formation of a stem-loop structure in a viral RNA genome can facilitate polymerase slippage [45], a duplication eventually occurred in both the DR(1+2) and DR3 regions. These duplications improved the fitness of the Asian CHIKV strain in *A. aegypti* to an extent that outweighed possible fitness loss in humans, and the duplicated mutant therefore rapidly replaced the previous 3′UTR to become fixed in Asia. This adaptation to the mosquito vector may have facilitated the initiation of Asian epidemics in 1958.

### Functional constraints and forces driving 3′UTR evolution

Studies in flaviviruses have provided important insights on the structure, evolution and functional importance of arboviral 3′UTRs. Basically, sequence duplications and deletions, in contrast to point mutations that are predominant in ORFs, are the major evolution mechanisms of flavivirus 3′UTRs [12]. Interestingly, the ORF region adjacent to the flavivirus 3′UTR [13], as well as 5′UTR panhandle structure [46], may also have originated from duplications of 3′ long repeat sequences (LRS). Furthermore, conserved secondary structures have been observed in the 3′UTRs of all eco-groups of flaviviruses [14]. However, despite the conservation of these DRs, there is no obvious relationship between them and secondary RNA structures; thus it is not clear why they are preserved, why they retain their double or triple copy numbers, and what exactly are their biological roles.

Similar to most previous studies with other viruses [3]–[8] except those of the flavivirus genome cyclization motif [47], [48], CHIKVs with deletions of different 3′UTR DRs remain infectious, although they exhibit a spectrum of replication reduction in C6/36 cells. The significant fitness differences in C6/36 cells but similar replication kinetics of these deletion mutants in Vero cells suggests that the 3′UTR plays a more important role in interacting with mosquito cell factors, and adaptation to vectors may be a major driving force for the evolution of the CHIKV 3′UTR. The importance of the 3′UTR in mosquito transmission is also supported by our findings of a strong impact on mosquito infection and dissemination caused by viruses with swapped 3′UTRs. In addition, although competition tests between different virus groups in CD1 mice suggested only minor fitness differences caused by the 3′UTR, CHIKVs with shorter genome lengths consistently outcompeted those with longer genomes in the vertebrate model (Fig. 3). Therefore, it is not clear if the higher fitness in the vertebrate host is due to an enhanced functional role of the 3′UTR or simply faster replication rates of shorter genomes.

Taken together, our results suggest that adaptation to mosquitoes is a major factor driving evolution of the CHIKV 3′UTR. This conclusion is in agreement with those form studies of flaviviruses. Deletion of the entire variable 3′UTR region of tick-borne encephalitis virus, but not the core element at its end, has no effect on BHK cell replication or murine virulence [6]. Interestingly, the longer 3′UTR favored for replication in arthropods has been taken to an extreme by Kamiti River (KRV) viruses, a flavivirus found only in mosquitoes and which cannot infect vertebrates. KRV, which contains a 3′UTR of 1208 nt that apparently resulted from self-duplication [49], suggests a major role for the 3′UTR for replication in insect cells. Likewise, the alphavirus Eilat, which also is restricted to insect cell infection, has a large 3′UTR of 520 nt [50]. In contrast, alphaviruses not known to be transmitted by vectors have very short 3′UTRs, including salmon pancreas disease virus (89 nt), sleeping disease virus (87 nt) [51], and salmonid alphavirus-3 with 87 nt [52].

However, the role of the 3′UTR in vertebrate cells should not be neglected entirely. For example, it is known that the 3′UTR affects alphavirus RNA stability in both mammalian and mosquito cells [17], [18]. Short deletions of different parts of the SINV 3′UTR lead to host-dependent fitness changes in mammalian, chicken and mosquito cells, suggesting that they are involved in interactions with different host-specific cellular factors [4]. In many cases a SINV 3′UTR deletion mutant is more severely impaired in mosquito than in chicken cells, but the inverse phenotype has also been observed [3]. A similar pattern is seen in dengue-4 virus, where deletion of a long upstream region (∼120 nt) of the 3′UTR leads to increased replication in simian LLC-MK_2_ cells but similar antibody responses in Rhesus monkeys, while other 3′UTR deletions reduce infectivity in both systems [5]. The balance between functional gain and reduced replication efficiency due to genome size may be key in determining the evolution of the 3′UTR.

### Mechanism of viral-host interaction and the differential effects on mammalian versus insect cells

What remains obscure is the exact nature of the molecular interaction mechanisms between arboviral 3′UTRs and cellular proteins, which have been proposed to be mediated by the stem-loop RNA [12]. Flavivirus studies [53] suggested that the level of perturbation of these secondary RNA structures rather than the size of deletions might affect viral replication. Our RNA secondary structural estimations suggest that duplication of CHIKV DR3 provides additional secondary structure, including the 80 nt conserved Y-shaped structure, without significantly changing other 3′UTR structures. Also, the enhanced replication in mosquitoes of CHIKV with this insertion suggests that this Y-shaped structure interacts with mosquito factors. However, the repeated elements DR1 and DR2 do not correspond strictly to structural units (Fig. S3), although the two copies of DR1 in the West Africa lineage retain the same predicted structure. Rather, duplication is predicted to result in the formation of new, local stem-loop structures, and more complicated secondary structures on a larger scale at 37°C.

The interaction of the cellular HuR protein with different alphaviruses (SINV, CHIKV, and Ross River virus) via different 3′UTR binding sites, probably all through AU rich sequences [18], suggests that the DR1 and DR2 region may also interact with cellular factors via primary sequence. Moreover, the stem-loop-rich structures in arboviral 3′UTRs may encode viral miRNAs. The recent discovery of an miRNA generated from the West Nile virus 3′UTR in infected mosquito cells, as well as the discovery of its host cellular target [20], provides evidence that viral miRNA can be important determinants of virus-host interactions. To explore the possibility of CHIKV-produced miRNA, we estimated the potential pri-miRNA sites in its genome using Vmir [54] and found some, including several in the 3′UTR (data not shown). Further experimental studies should be carried out to confirm these predictions.

The significant effect of genetic change in CHIKV 3′UTR on the fitness level in mosquito and mosquito cells, but not in vertebrate cells, could reflect an interaction between CHIKV and insect-specific genes or proteins, such as those in the antiviral RNA interference (RNAi) pathway, the major insect innate immune mechanism [55]. This hypothesis is supported by the flavivirus sfRNA's role in RNA inference suppression in both mammalian and insect cells by inhibiting Dicer-mediated in vitro cleavage of double-stranded RNA [27]. Another possibility is that the 3′UTR may fold into different structures in vertebrate vs. mosquito cells maintained at different temperatures, as suggested by our Mfold results. This structural difference could affect protein binding. In addition, the presence of a miRNA (WNV) generated in mosquito but not mammalian cells suggests that the miRNA processing may differ between these cell types [20]. Finally, mammalian cells may have more redundant gene expression regulation systems where the effect of down-regulation in one signal transduction pathway can be compensated via other intertwined pathways, making them more robust in their viral regulation of gene expression than insect cells.

In conclusion, we observed for the first time lineage-specific evolution of the 3′UTR in an arbovirus, and our results suggest that the CHIKV 3′UTR plays an important role in adaptation to the mosquito vector. The founder effect that our results suggest was apparently responsible for the establishment of an inferior CHIKV 3′UTR in Asia before or during the 1950s. This reinforces our previous findings, which demonstrated epistatic mutations in this CHIKV lineage that probably resulted from the same founder effect, and which limited the fitness and adaptation of Asian strains [56].

## Materials and Methods

### Ethics statement

This study was carried out in strict accordance with the recommendations in the Guide for the Care and Use of Laboratory Animals of the National Institutes of Health. The protocol # 02-09-068 was approved by the Institutional Animal Care and Use Committee of the University of Texas Medical Branch.

### Sequence alignment and repeat pattern identification

All available complete genome sequences of CHIKV were downloaded from the GenBank library. A maximum Likelihood tree was constructed based on the complete coding sequences (CDS) using PAUP* v4.0b [57], utilizing the best-fit model estimated by MODELTEST [58]. This ML tree was then used as a guide to generate a sequence alignment of 3′UTR of CHIKV utilizing MAFFT [59]. Strains with incomplete 3′UTRs and those with unique indel patterns were excluded, leading to a dataset of 108 sequences. Sequence repeats were identified using the DNA matrix analysis in MacVector® based on representative strains of each lineage, followed by manual adjustments based on sequence alignments.

### RNA secondary structure estimation

To determine whether DRs form structural units, the RNA secondary structure of the CHIKV 3′UTR from each lineage was estimated using several programs with different advantages, including Mfold [32], STAR (STructure Analysis of Rna; 33) and Vienna RNA Secondary Structure Package [34], [35], based on either representative strains (Mfold and STAR) or a sequence alignment of the entire lineage (RNAalifold in Vienna). Mfold provides a selection of top rated structures, which are all plausible given the dynamic movement of molecules. The Vienna RNAalifold program is based on sequence alignment rather than a single sequence, thus reflecting the natural selection on the secondary structure. The “Genetic Algorithm” implemented in STAR uses the procedure of stepwise selection of the most-fit structures, which simulates the process of RNA elongation during synthesis, allowing the observation of important intermediate structures. The resulted structures were illustrated using PseudoViewer webserver [60].

### Construction of infectious cDNA clones

Plasmids representing two wt CHIKV strains (Mal06 and SL07) from the Asian and ECSA lineages, respectively, have been described previously [56]. The Mal06 strain (MY002IMR/06/BP; GenBank Acc. No. EU703759.1), a representative of the Asian lineage, was isolated from a human in Bagan Panchor, Malaysia in 2006. Its cDNA was synthesized directly from GenBank sequence. The SL07 strain (SL-CK1; GenBank Acc. No. HM045801.1), isolated from a human during the Sri Lanka outbreak in 2007, represents the ECSA lineage, and was passed twice in C6/36 mosquito cells before cDNA synthesis from viral RNA. These two plasmids were used for rescuing wt viruses and as templates to generate other infectious clones with altered 3′UTRs. To study the genetic change of the 3′UTR in the Asian lineage, a series of deletions in the direct repeats DR(1+2) and DR3 was constructed based on conventional PCR methods [61]. Similarly, chimeric viruses with the backbones of the Mal06 and SL07 strains and swapped 3′UTRs were also constructed to compare the fitness of the lineage-specific 3′UTRs. Finally, to compare the relative fitness levels of two viruses using a competition test (described below), a synonymous point mutation was introduced into both Mal06 and SL07 plasmids in the nsP4 gene to form a restriction digestion site cleavable by endonucleases *Apa* I and *Psp*OMI [56]. All PCR-generated genome regions used in cloning were completely sequenced to verify their genetic integrity. Detailed information for all plasmids is available from the authors upon request.

### Rescue of viruses from the infectious clone

To generate infectious RNA, plasmids were first linearized with *Not* I restriction endonuclease, followed by *in vitro* transcription from the minimal SP6 promoter as described previously [61]. About 10 micrograms of RNA were electroporated into 10^7^ BHK-21 cells (viruses with Mal06 backbone) or C7/10 cells (viruses with SL07 backbone) using the BTX-Harvard Apparatus ECM 830 Square Wave Electroporator (Harvard Apparatus) and 2-mm or 4-mm cuvette, respectively. Cell culture supernatants were harvested between 24 and 48 h post-electroporation and stored at −80°C. Infectious viral titers were determined by titration on Vero cells by plaque assay (range from ×10^6^ to ×10^8^ pfu/ml, data not shown). All viruses recovered from electroporation were used directly without any additional passages.

### Replication in cell culture

Forty-eight hour replication curves were performed in C6/36 mosquito (*A. albopictus*) and Vero (African green monkey) cells in triplicate. Cell monolayers at 80% confluency in T25 flasks were incubated with 1 mL of viral suspension at an approximate multiplicity of infection (MOI) of 0.1 PFU/cell for 1 h at 37°C (Vero) or 28°C (C6/36). After the incubation, the monolayers were washed thrice with PBS and then incubated in the corresponding cell growth medium. Aliquots of the supernatant were harvested and replaced at each of the following time points: 0 h, 4 h, 8 h, 12 h, 24 h, 48 h post-infection. The viral RNA levels were evaluated by Real-time RT-PCR using primers, probes and cycle conditions available from the authors.

### Competition experiments

To sensitively determine the relative fitness levels of mutant vs. wt viruses, competition tests were conducted in mosquitos and mice as described previously [56]. The competing viruses, with and without the genetic marker, were mixed in a 1∶1 ratio and then inoculated into mice or mosquitoes. Viral RNA was purified from each sample as well as the initial inoculation stock using the QiAamp Viral RNA kit (QIAGEN). To evaluate the ratio of the two viruses from total viral RNA, the genome region from nt 6106–6794, which covers the position of the introduced ApaI/PspOMI digestion site in the middle, was amplified by RT-PCR using QIAGEN OneStep RT-PCR kit (QIAGEN) using the following primers: 41855-nsF5: 5′-ATATCTAGACATGGTGGA-3′; 41855-nsR1: 5′-TATCAAAGGAGGCTATGTC-3′. The PCR products were subject to digestion by restriction endonucleases Apa I and *Psp*OMI (sharing the same cutting site) for 30 min at 27°C, 4 h at 37°C. Complete digestion was confirmed using controls, which contained only one of the competing viruses (with or without the marker).

### Fitness in mosquitoes

The relative replication and dissemination rates in mosquito vectors of competing viruses were examined in an *A. aegypti* colony established from mosquito eggs collected in 2009 in Bangkok, Thailand. The competing viruses were mixed with sheep blood to a final concentration of ∼1×10^6^ pfu/mL. Mosquitoes collected 4–5 days after eclosion were offered blood meals for ∼45 min, and engorged mosquitoes were sorted and incubated at 28°C with 10% sucrose and 80% relative humidity under a 16 h light/8 h dark photoperiod. At 10 day post-infection (dpi), heads of 60 mosquitoes were dissected and homogenized in 1.5 ml of MEM. Because the titers of head suspensions were too low for consistent RT-PCR amplification, viruses from these samples were amplified by infecting Vero cells prepared in 96-well plates. Supernatants from Vero cells showing CPE were collected at 2 dpi and used for RNA extraction, RT-PCR and digestion, as well as gel analyses as described above. To ensure that major differences in fitness for replication in Vero cells did not affect these mosquito fitness assays, each mutant was competed first in Vero cells. Except for Mal06/ΔDR(1+2)a, there was no detectable difference in fitness for any of the mutants compared to wt (Fig. S4B). Mal06/ΔDR(1+2)a did have a moderate fitness advantage for replication in Vero cells (Fig. S4B), but this, difference was contrary to the results of mosquito competitions (where Mal06/ΔDR(1+2)a consistently lost). Therefore, the Vero cell passages could not have confounded the mosquito competition results.

### Fitness in mice

About ten 11 to 12-day-old outbred CD1mice (Charles River) were subcutaneously infected with 50 µL of virus mixtures containing a total of ∼1000 pfu. Three or four mice were sacrificed daily from day 1 to 3 dpi and blood samples were collected for subsequent RNA extraction and genetic analyses as described above.

## Supporting Information

Figure S1 **DNA matrix comparison of different lineages (ECSA, Asia, and West Africa) of CHIKV.** One representative strain of each lineage was used to compare with itself in search of repeating structure in the genome. Left plots are based on whole genomes. Right plots are based on the 3′UTR only of the corresponding strain. Lines parallel to the central diagonal line suggest direct repeats, whereas lines perpendicular (not found) to the central diagonal line suggests reverse repeats.
(EPS)

Figure S2 **Sequence alignment of CHIKV 3′UTR.** Alignment of 33 CHIKV 3′UTR sequences, showing the majority of genetic diversity, present in the 108 strain sequence alignment. Sequences are arranged by lineages with names and lineage shown on the left. Direct repeats are indicated by rectangular blocks in different colors superimposed on the sequence alignment.
(TIF)

Figure S3 **Secondary structures of CHIKV 3′UTR in each lineage based on Mfold.** Examples of top structures estimated from Mfold at both 37°C (upper panel) and 28°C (lower panel). Direct repeats are mapped on structures using the same colors corresponding to Fig. 1. A 80 nt conserved Y-shaped structure is colored in dark blue within Direct Repeat 3.
(EPS)

Figure S4 **Competition tests on Vero cells.** Four competition pairs (A: Mal06/ΔDR3a vs. Mal06/WT+marker; B: Mal06/ΔDR(1+2)a vs. Mal06/WT+marker; C: Mal06/SL07-3′UTR vs. Mal06/WT+marker; D: SL07/Mal06-3′UTR vs. SL07/WT+marker) were tested. For each competition pair, two viruses were mixed in 1∶1 RNA ratio and inoculated to three T25 flasks of monolayer Vero cells with MOI = 0.1. The supernatant was harvested in 48 hours post infection and then subjected to RNA purification, RT-PCR and digestion by ApaI and *Psp*OMI. The genetically marked virus is represented by the lower band, and the unmarked by the lower band.
(EPS)

## References

[1] Tsetsarkin KA, Vanlandingham DL, McGee CE, Higgs S (2007) A single mutation in chikungunya virus affects vector specificity and epidemic potential. PLoS Pathog 3: e201. 18069894 10.1371/journal.ppat.0030201 PMC2134949

[2] Brault AC, Huang CY, Langevin SA, Kinney RM, Bowen RA, et al (2007) A single positively selected West Nile viral mutation confers increased virogenesis in American crows. Nat Genet 39: 1162–1166. 17694056 10.1038/ng2097 PMC2291521

[3] Kuhn RJ, Hong Z, Strauss JH (1990) Mutagenesis of the 3′ nontranslated region of Sindbis virus. RNA J Virol 64: 1465–1476. 2319643 10.1128/jvi.64.4.1465-1476.1990 PMC249280

[4] Kuhn RJ, Griffin DE, Zhang H, Niesters HJ, Strauss JH (1992) Attenuation of Sindbis virus neurovirulence by using defined mutations in nontranslated regions of the genome. RNA J Virol 66: 7121–7127. 1433509 10.1128/jvi.66.12.7121-7127.1992 PMC240395

[5] Men R, Bray M, Clark D, Chanock RM, Lai CJ (1996) Dengue type 4 virus mutants containing deletions in the 30 noncoding region of the RNA genome: Analysis of growth restriction in cell culture and altered viremia pattern and immunogenicity in rhesus monkeys. J Virol 70: 3930–3937. 8648730 10.1128/jvi.70.6.3930-3937.1996 PMC190271

[6] Mandl CW, Holzmann H, Meixner T, Rauscher S, et al (1998) Spontaneous and engineered deletions in the 3′ noncoding region of tick-borne encephalitis virus: construction of highly attenuated mutants of a flavivirus. J Virol 72: 2132–2140. 9499069 10.1128/jvi.72.3.2132-2140.1998 PMC109508

[7] Lo MK, Tilgner M, Bernard KA, Shi PY (2003) Functional analysis of mosquito-borne flavivirus conserved sequence elements within 3′ untranslated region of West Nile virus by use of a reporting replicon that differentiates between viral translation and RNA replication. J Virol 77: 10004–10014. 12941911 10.1128/JVI.77.18.10004-10014.2003 PMC224605

[8] George J, Raju R (2000) Alphavirus RNA genome repair and evolution: molecular characterization of infectious sindbis virus isolates lacking a known conserved motif at the 3′ end of the genome. J Virol 74: 9776–9785. 11000254 10.1128/jvi.74.20.9776-9785.2000 PMC112414

[9] Ou JH, Trent DW, Strauss JH (1982) The 3′-non-coding regions of alphavirus RNAs contain repeating sequences. J Mol Biol 156: 719–730. 6288962 10.1016/0022-2836(82)90138-3

[10] Pfeffer M, Kinney RM, Kaaden OR (1998) The alphavirus 3′-nontranslated region: size heterogeneity and arrangement of repeated sequence elements. Virology 240: 100–108. 9448694 10.1006/viro.1997.8907

[11] Gritsun TS, Gould EA (2006) Direct repeats in the 3′ untranslated regions of mosquito-borne flaviviruses: possible implications for virus transmission. J Gen Virol 87: 3297–3305. 17030864 10.1099/vir.0.82235-0

[12] Gritsun TS, Gould EA (2007) Origin and evolution of 3′UTR of flaviviruses: long direct repeats as a basis for the formation of secondary structures and their significance for virus transmission. Adv Virus Res 69: 203–248. 17222695 10.1016/S0065-3527(06)69005-2

[13] Gritsun TS, Gould EA (2006) The 3′ untranslated region of tick-borne flaviviruses originated by the duplication of long repeat sequences within the open reading frame. Virology 350: 269–275. 16730048 10.1016/j.virol.2006.03.002

[14] Proutski V, Gould EA, Holmes EC (1997) Secondary structure of the 3′ untranslated region of flaviviruses: similarities and differences. Nucleic Acids Res 25: 1194–1202. 9092629 10.1093/nar/25.6.1194 PMC146583

[15] Pardigon N, Lenches E, Strauss JH (1993) Multiple binding sites for cellular proteins in the 3′ end of Sindbis alphavirus minus-sense RNA. J Virol 67: 5003–5011. 8392625 10.1128/jvi.67.8.5003-5011.1993 PMC237888

[16] Garneau NL, Sokoloski KJ, Opyrchal M, Neff CP, Wilusz CJ, et al (2008) The 3′ untranslated region of sindbis virus represses deadenylation of viral transcripts in mosquito and Mammalian cells. J Virol 82: 880–892. 17977976 10.1128/JVI.01205-07 PMC2224598

[17] Sokoloski KJ, Dickson AM, Chaskey EL, Garneau NL, Wilusz CJ, et al (2010) Sindbis virus usurps the cellular HuR protein to stabilize its transcripts and promote productive infections in mammalian and mosquito cells. Cell Host Microbe 8: 196–207. 20709296 10.1016/j.chom.2010.07.003 PMC2929003

[18] Dickson AM, Anderson JR, Barnhart MD, Sokoloski KJ, Oko L, et al (2012) Dephosphorylating of HuR protein during alphavirus infection is associated with HuR relocalization to the cytoplasm. J Biol Chem 287: 36229–36238. 22915590 10.1074/jbc.M112.371203 PMC3476290

[19] Lei Y, Huang Y, Zhang H, Yu L, Zhang M, Dayton A (2011) Functional interaction between cellular p100 and the dengue virus 3′ UTR. J Gen Virol 92: 796–806. 21148275 10.1099/vir.0.028597-0

[20] Hussain M, Torres S, Schnettler E, Funk A, Grundhoff A, et al (2012) West Nile virus encodes a microRNA-like small RNA in the 3′ untranslated region which up-regulates GATA4 mRNA and facilitates virus replication in mosquito cells. Nucleic Acids Res 40: 2210–2023. 22080551 10.1093/nar/gkr848 PMC3300009

[21] Pijlman GP, Funk A, Kondratieva N, Leung J, Torres S, et al (2008) A highly structured, nuclease-resistant, noncoding RNA produced by flaviviruses is required for pathogenicity. Cell Host Microbe 4: 579–591. 19064258 10.1016/j.chom.2008.10.007

[22] Silva PA, Pereira CF, Dalebout TJ, Spaan WJ, Bredenbeek PJ (2010) An RNA pseudoknot is required for production of yellow fever virus subgenomic RNA by the host nuclease XRN1. J Virol 84: 11395–11406. 20739539 10.1128/JVI.01047-10 PMC2953177

[23] Funk A, Truong K, Nagasaki T, Torres S, Floden N, et al (2010) RNA structures required for production of subgenomic flavivirus RNA. J Virol 84: 11407–11417. 20719943 10.1128/JVI.01159-10 PMC2953152

[24] Fan YH, Nadar M, Chen CC, Weng CC, Lin YT, et al (2011) Small noncoding RNA modulates Japanese encephalitis virus replication and translation in trans. Virol J 8: 492. 22040380 10.1186/1743-422X-8-492 PMC3221644

[25] Moon SL, Anderson JR, Kumagai Y, Wilusz CJ, Akira S, et al (2012) A noncoding RNA produced by arthropod-borne flaviviruses inhibits the cellular exoribonuclease XRN1 and alters host mRNA stability. RNA 18: 2029–2040. 23006624 10.1261/rna.034330.112 PMC3479393

[26] Schuessler A, Funk A, Lazear HM, Cooper DA, Torres S, et al (2012) West Nile virus noncoding subgenomic RNA contributes to viral evasion of the type I interferon-mediated antiviral response. J Virol 86: 5708–5718. 22379089 10.1128/JVI.00207-12 PMC3347305

[27] Schnettler E, Sterken MG, Leung JY, Metz SW, Geertsema C, et al (2012) Noncoding flavivirus RNA displays RNA interference suppressor activity in insect and Mammalian cells. J Virol 86: 13486–13500. 23035235 10.1128/JVI.01104-12 PMC3503047

[28] Volk SM, Chen R, Tsetsarkin KA, Adams AP, Garcia TI, et al (2010) Genome-scale phylogenetic analyses of chikungunya virus reveal independent emergences of recent epidemics and various evolutionary rates. J Virol 84: 6497–6504. 20410280 10.1128/JVI.01603-09 PMC2903258

[29] Burt FJ, Rolph MS, Rulli NE, Mahalingam S, Heise MT (2012) Chikungunya: a re-emerging virus. Lancet 379: 662–671. 22100854 10.1016/S0140-6736(11)60281-X

[30] Wolfe ND, Kilbourn AM, Karesh WB, Rahman HA, Bosi EJ, et al (2001) Sylvatic transmission of arboviruses among Bornean orangutans. Am J Trop Med Hyg 64: 310–316. 11463123 10.4269/ajtmh.2001.64.310

[31] Hardy RW, Rice CM (2005) Requirements at the 3′ end of the sindbis virus genome for efficient synthesis of minus-strand RNA. J Virol 79: 4630–4639. 15795249 10.1128/JVI.79.8.4630-4639.2005 PMC1069581

[32] Zuker M (2003) Mfold web server for nucleic acid folding and hybridization prediction. Nucleic Acids Res 31: 3406–3415. 12824337 10.1093/nar/gkg595 PMC169194

[33] van Batenburg FHD, Gultyaev AP, Pleij CWA (1995) An APL-programmed Genetic Algorithm for the Prediction of RNA Secondary Structure. J Theor Biol 174: 269–280. 7545258 10.1006/jtbi.1995.0098

[34] Hofacker IL, Fekete M, Stadler PF (2002) Secondary Structure Prediction for Aligned RNA Sequences. J Mol Biol 319: 1059–1066. 12079347 10.1016/S0022-2836(02)00308-X

[35] Bernhart SH, Hofacker IL, Will S, Gruber AR, Stadler PF (2008) RNAalifold: Improved consensus structure prediction for RNA alignments. BMC Bioinformatics 9: 474. 19014431 10.1186/1471-2105-9-474 PMC2621365

[36] Hapuarachchi HC, Bandara KB, Sumanadasa SD, Hapugoda MD, Lai YL, et al (2010) Re-emergence of Chikungunya virus in South-east Asia: virological evidence from Sri Lanka and Singapore. J Gen Virol 91: 1067–1076. 19955565 10.1099/vir.0.015743-0

[37] Ziegler SA, Lu L, da Rosa AP, Xiao SY, Tesh RB (2008) An animal model for studying the pathogenesis of chikungunya virus infection. Am J Trop Med Hyg 79: 133–139. 18606777

[38] Proutski V, Gaunt MW, Gould EA, Holmes EC (1997) Secondary structure of the 3′-untranslated region of yellow fever virus: implications for virulence, attenuation and vaccine development. J Gen Virol 78: 1543–1549. 9225027 10.1099/0022-1317-78-7-1543

[39] Bryant JE, Vasconcelos PF, Rijnbrand RC, Mutebi JP, Higgs S, et al (2005) Size heterogeneity in the 3′ noncoding region of South American isolates of yellow fever virus. J Virol 79: 3807–3821. 15731274 10.1128/JVI.79.6.3807-3821.2005 PMC1075708

[40] Troyer JM, Hanley KA, Whitehead SS, Strickman D, Karron RA, et al (2001) A live attenuated recombinant dengue-4 virus vaccine candidate with restricted capacity for dissemination in mosquitoes and lack of transmission from vaccinees to mosquitoes. Am J Trop Med Hyg 65: 414–419. 11716092 10.4269/ajtmh.2001.65.414

[41] Najera JA (1989) Malaria and the work of WHO. Bull World Health Organ 67: 229–243. 2670294 PMC2491250

[42] Brown A (2002) Personal experiences in the malaria eradication campaign 1955–1962. J R Soc Med 95: 154–156. 11872773 10.1258/jrsm.95.3.154 PMC1279490

[43] Severo OP (1956) Aedes agypti eradication in the Americas. WHO Chron 10: 347–354. 14075868

[44] Camargo S (1967) History of Aedes aegypti eradication in the Americas. Bull Wld Hlth Org 36: 602–603. PMC2476393 5299460

[45] Eckert KA, Hile SE (2009) Every microsatellite is different: Intrinsic DNA features dictate mutagenesis of common microsatellites present in the human genome. Mol Carcinog 48: 379–388. 19306292 10.1002/mc.20499 PMC2731485

[46] Gritsun TS, Gould EA (2007) Origin and evolution of flavivirus 5′UTRs and panhandles: trans-terminal duplications? Virology 366: 8–15. 17658577 10.1016/j.virol.2007.04.011

[47] Khromykh AA, Meka H, Guyatt KJ, Westaway EG (2001) Essential role of cyclization sequences in flavivirus RNA replication. J Virol 75: 6719–28. 11413342 10.1128/JVI.75.14.6719-6728.2001 PMC114398

[48] Bredenbeek PJ, Kooi EA, Lindenbach B, Huijkman N, Rice CM, et al (2003) A stable full-length yellow fever virus cDNA clone and the role of conserved RNA elements in flavivirus replication. J Gen Virol 84: 1261–1268. 12692292 10.1099/vir.0.18860-0

[49] Gritsun TS, Gould EA (2006) The 3′ untranslated regions of Kamiti River virus and Cell fusing agent virus originated by self-duplication. J Gen Virol 87: 2615–2619. 16894200 10.1099/vir.0.81950-0

[50] Nasar F, Palacios G, Gorchakov RV, Guzman H, Da Rosa AP, et al (2012) Eilat virus, a unique alphavirus with host range restricted to insects by RNA replication. Proc Natl Acad Sci U S A 109: 14622–14627. 22908261 10.1073/pnas.1204787109 PMC3437828

[51] Weston J, Villoing S, Brémont M, Castric J, Pfeffer M, et al (2002) Comparison of two aquatic alphaviruses, salmon pancreas disease virus and sleeping disease virus, by using genome sequence analysis, monoclonal reactivity, and cross-infection. J Virol 76: 6155–6163. 12021349 10.1128/JVI.76.12.6155-6163.2002 PMC136221

[52] Karlsen M, Villoing S, Rimstad E, Nylund A (2009) Characterization of untranslated regions of the salmonid alphavirus 3 (SAV3) genome and construction of a SAV3 based replicon. Virol J 6: 173. 19860871 10.1186/1743-422X-6-173 PMC2772843

[53] Proutski V, Gritsun TS, Gould EA, Holmes EC (1999) Biological consequences of deletions within the 3′-untranslated region of flaviviruses may be due to rearrangements of RNA secondary structure. Virus Res 64: 107–123. 10518708 10.1016/s0168-1702(99)00079-9

[54] Sullivan CS, Grundhoff A (2007) Identification of viral miRNAs. Meth Enzymol 427: 3–23. 17720476 10.1016/S0076-6879(07)27001-6

[55] Blair CD (2011) Mosquito RNAi is the major innate immune pathway controlling arbovirus infection and transmission. Future Microbiol 6: 265–277. 21449839 10.2217/fmb.11.11 PMC3126673

[56] Tsetsarkin KA, Chen R, Leal G, Forrester N, Higgs S, et al (2011) Chikungunya virus emergence is constrained in Asia by lineage-specific adaptive landscapes. Proc Natl Acad Sci U S A 108: 7872–7877. 21518887 10.1073/pnas.1018344108 PMC3093459

[57] Swofford DL. (2003) PAUP*. Phylogenetic analysis using parsimony (*and other methods). Version 4. Sunderland (MA): Sinauer Associates.

[58] Posada D, Crandall KA (1998) Modeltest: testing the model of DNA substitution. Bioinformatics 14: 817–818. 9918953 10.1093/bioinformatics/14.9.817

[59] Katoh K, Asimenos G, Toh H (2009) Multiple Alignment of DNA Sequences with MAFFT. Methods Mol Biol 537: 39–64. 19378139 10.1007/978-1-59745-251-9_3

[60] Byun Y, Han K (2009) PseudoViewer3: generating planar drawings of large-scale RNA structures with pseudoknots,. Bioinformatics 25: 1435–1437. 19369500 10.1093/bioinformatics/btp252

[61] Tsetsarkin K, Higgs S, McGee CE, De Lamballerie X, Charrel RN, et al (2006) Infectious clones of Chikungunya virus (La Réunion isolate) for vector competence studies. Vector Borne Zoonotic Dis 6: 325–337. 17187566 10.1089/vbz.2006.6.325

