# Supplementary figures and images for: Chikungunya Virus 3′ Untranslated Region: Adaptation to Mosquitoes and a Population Bottleneck as Major Evolutionary Forces
Source: PLoS Pathog. 2013 Aug 29;9(8):e1003591. doi: 10.1371/journal.ppat.1003591 (PMC3757053; doi:10.1371/journal.ppat.1003591)

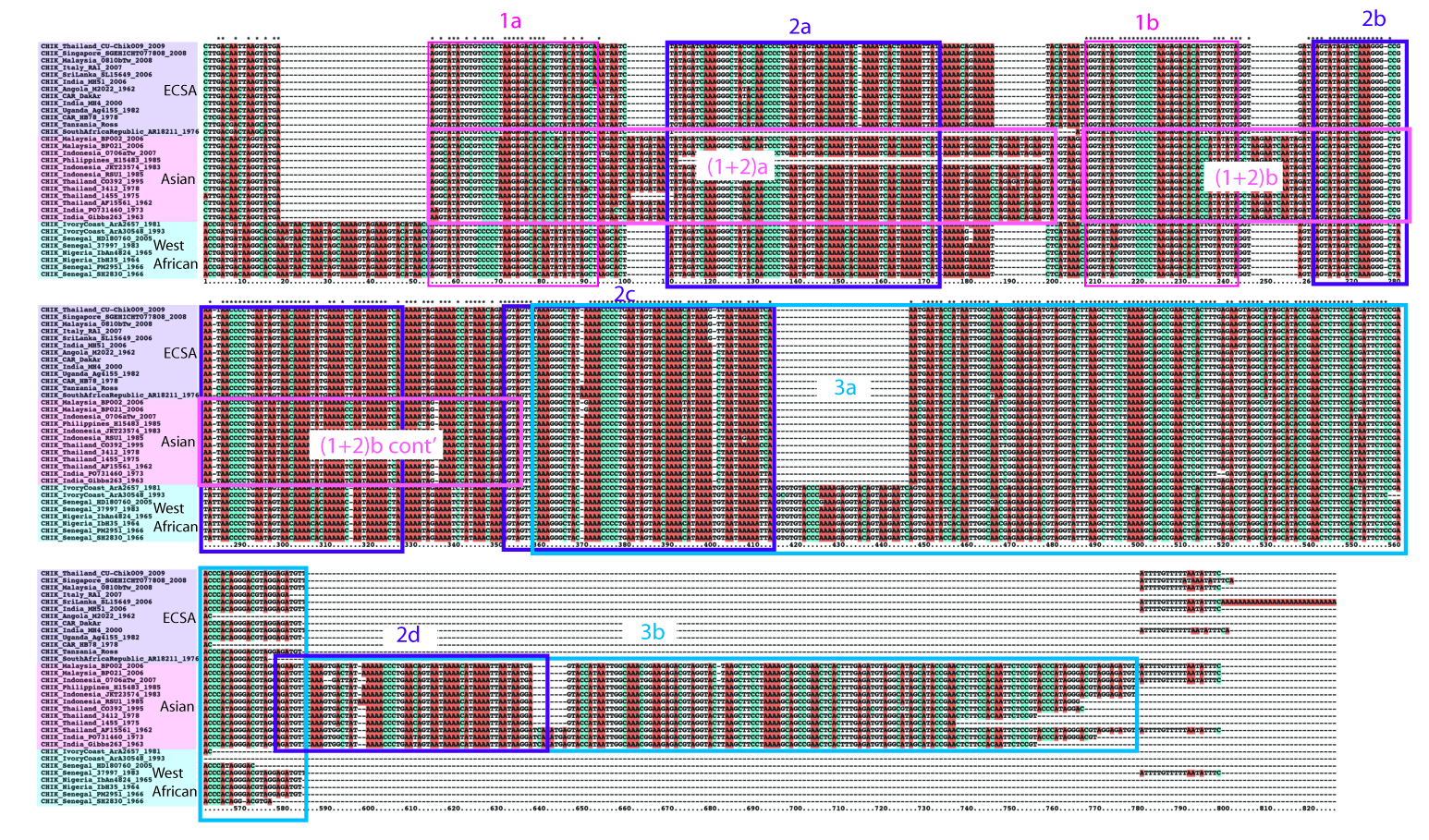

Supplement: Figure S2 — Sequence alignment of CHIKV 3′UTR. Alignment of 33 CHIKV 3′UTR sequences, showing the majority of genetic diversity, present in the 108 strain sequence alignment. Sequences are arranged by lineages with names and lineage shown on the left. Direct repeats are indicated by rectangular blocks in different colors superimposed on the sequence alignment. (TIF) [file ppat.1003591.s002.tif]
